# Robust Silica-Cellulose Composite Aerogels with a Nanoscale Interpenetrating Network Structure Prepared Using a Streamlined Process

**DOI:** 10.3390/polym12040807

**Published:** 2020-04-03

**Authors:** Huazheng Sai, Jing Zhang, Zhiqiang Jin, Rui Fu, Meijuan Wang, Yutong Wang, Yaxiong Wang, Litong Ma

**Affiliations:** 1 School of Chemistry and Chemical Engineering, Inner Mongolia University of Science & Technology, Baotou 014010, China; shz15@tsinghua.org.cn (H.S.);; 2 Inner Mongolia Engineering Research Center of Comprehensive Utilization of Bio-coal Chemical Industry, Baotou 014010, China; 3 Inner Mongolia Key Laboratory of Coal Chemical Engineering & Comprehensive Utilization, Baotou 014010, China

**Keywords:** silica aerogels, bacterial cellulose, nanocomposites, interpenetrating network, bendable, mechanical properties

## Abstract

Silica aerogels can be strengthened by forming a nanoscale interpenetrating network (IPN) comprising a silica gel skeleton and a cellulose nanofiber network. Previous studies have demonstrated the effectiveness of this method for improving the mechanical properties and drying of aerogels. However, the preparation process is generally tedious and time-consuming. This study aims to streamline the preparation process of these composite aerogels. Silica alcosols were directly diffused into cellulose wet gels with loose, web-like microstructures, and an IPN structure was gradually formed by regulating the gelation rate. Supercritical CO_2_ drying followed to obtain composite aerogels. The mechanical properties were further enhanced by a simple secondary regulation process that increased the quantity of bacterial cellulose (BC) nanofibers per unit volume of the matrix. This led to the production of aerogels with excellent bendability and a high tensile strength. A maximum breaking stress and tensile modulus of 3.06 MPa and 46.07 MPa, respectively, were achieved. This method can be implemented to produce robust and bendable silica-based composite aerogels (CAs).

## 1. Introduction

Aerogels are synthetic materials characterized by fine internal void spaces, open-pore geometry, and useful properties including low density, high porosity, high specific surface, and low thermal conductivity [1,2,3]. These materials have broad application potential in thermal insulation [4,5], oil absorption [6], catalysis [7], electrode materials [8], CO_2_ remove [9], tissue engineering [10], energy storage [11], adsorption of heavy metal ions [12], and as drug carriers [13]. The unique microstructure of aerogels is associated with low solids content and a fragile gel skeleton that cannot withstand external impact, and typical inorganic oxide aerogels with a pearl-necklace-like gel skeleton are particularly vulnerable [14,15]. The weak mechanical strength of aerogels means that they must be carefully manufactured, and these tedious and time-consuming preparation processes are often costly [16]. Reinforcing aerogels is a simple approach to address these issues, allowing for practical application.

Silica aerogels are typical inorganic oxide aerogels that have been widely studied to improve their mechanical properties and streamline the preparation process [17,18]. Two strategies have been explored: the construction of an organic–inorganic hybrid gel skeleton [19,20] and the introduction of reinforcing materials in the gel skeleton to produce a composite aerogel [21,22]. Hybrid gel skeleton construction involves the synthesis of a special precursor with a flexible group or chain segment through an innovative organic reaction, followed by the sol-gel process [23,24]. The precursor is expensive and increases the cost of the aerogel. Various reinforcing materials such as ceramic fibers [25], polymer fibers [26,27], glass fibers [28], and a network-like matrix comprising these fibers [29] were used as structural strengthening agents in earlier studies on composite silica aerogels. These composite aerogels are associated with substantial dust release, as relatively macroscopic structural strengthening agents are not dimensionally compatible with the nanoscale silica gel skeleton. The above two strategies will also be used in combination to better improve the mechanical properties of silica aerogels [30]. More recently, conformal coating of the silica gel skeleton with a polymer [31] and the construction of a nanoscale interpenetrating network (IPN) [32,33,34] have been proposed. The conformal polymer coating method strengthens the gel skeleton and preserves the mesopores of silica aerogels. However, the micropores in the silica gel skeleton are susceptible to being filled by the polymer, which leads to an increase in density and a decrease in the surface area of the aerogel [35]. Constructing a silica gel skeleton in a polymer matrix involves a nanofiber network that forms a nanoscale IPN, which effectively addresses and improves the brittleness of silica aerogels. Rather than individually dispersed nanofibers, a web-like matrix with entangled and cross-linked ultrafine nanofibers can be used to further improve the mechanical properties [36]. This also preserves the microstructure of the pearl-necklace-like silica gel skeleton [37], and does not compromise the excellent physical properties of the silica aerogel.

**Figure 1:**
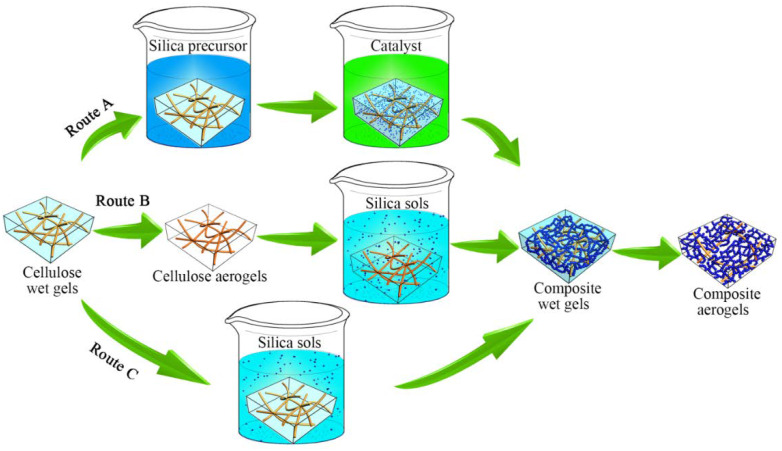
Schematic of the preparation process for composite aerogels with a nanoscale interpenetrating network (IPN) structure via three different routes.

Cellulose, as abundant native polymer, has gained recent popularity due to its renewable and environmentally-friendly properties [38,39], including enhanced aerogel strength [27]. Recent studies have combined a cellulose nanofiber network matrix with a silica gel skeleton to produce a nanoscale IPN structure [36,40,41,42,43]. The strength of these silica-cellulose composite aerogels was over two orders of magnitude higher than that of native silica aerogels. There are two main preparation methods for producing these composite aerogels, both of which are notably tedious and time-consuming. The first method involves preparing a cellulose wet gel matrix into which a silica precursor is diffused followed by a catalyst, resulting in a silica gel skeleton (route A in Figure 1) [41,44,45]. This two-step diffusion process is time-consuming, and the silica precursor at the surface of the matrix can separate from the matrix before gelation. The second method involves soaking a dried cellulose web-like nanofiber matrix (i.e., cellulose aerogels) in a prepared silica sol to complete the ongoing sol-gel reaction in the matrix (route B in Figure 1) [33,36,40,42,46]. While diffusion occurs in a single step, the matrix must be dried using freeze-drying or supercritical drying to preserve the web-like structure, both of which are tedious. A simpler process should be developed to save time and improve preparation efficiency.

This study aimed to use alcosols directly diffused in wet bacterial cellulose (BC) gel to gradually form an IPN structure in situ, as shown in route C in Figure 1. Firstly, it benefits from the fact that the BC nanofibers in the matrix were more loosely distributed and created gaps between the nanofibers measuring up to hundreds of nanometers. Then, the gelation rate was restricted using low temperature to ensure that the silica alcosols were sufficiently diffused into the wet BC matrix. The soft wet matrix with silica alcosols could be compressed before the rigid and fragile silica gel skeleton formed to increase the content of BC nanofibers per unit volume and regulate the mechanical properties of composite aerogels.

## 2. Materials and Methods

### 2.1. Materials

*Acetobacter xylinum* was obtained from the Shanghai Biological Conservation Center. Tetraethoxysilane (TEOS, 28% SiO_2_) was obtained from the Modern Orient Technology Development Co. Ltd. (Beijing, China), while sodium hydroxide (NaOH), hydrochloric acid (HCl), ammonia (NH_3_•H_2_O), ethanol (≥99.7%) were obtained from Beijing Chemical Reagents Co. (Beijing, China). All chemicals were used as received without further purification. 

### 2.2. Preparation of the Bacterial Cellulose (BC) Wet Gel (i.e., Matrix)

*Acetobacter xylinum* was grown on solid agar until visible colonies were formed. A single colony was expanded in liquid media for 3 days and transferred to an Erlenmeyer flask with 200 mL liquid media. A thin gel film of BC formed at the surface of liquid media over a period of 5 days (Figure S1a). The liquid culture media consisted of 20 g/L glucose, 5 g/L peptone, 5 g/L yeast extract, 1.15 g/L citric acid monohydrate, and 6.8 g/L Na_2_HPO_4_•12H_2_O. The produced BC hydrogel was heated to 90 °C in NaOH solution (6% *w/w*) for 2 h and washed with deionized water to neutral (Figure S1b).

### 2.3. Diffusion of Silica Alcosols in BC Wet Gel

A mixed solution of TEOS, 4 mL deionized water, 20 mL ethanol, and 0.4 mL HCl (1% *w/w*) was stirred evenly at room temperature for 1.5 h and cooled in an ice-water bath for 1 h. The amount of precursor TEOS used in the mixed solution was initially varied at 1.7, 3.4, 5.1 and 6.8 mL. Silica alcosols were formed by adding 2 mL dilute ammonia (0.1 mol L^−1^) to the mixed solution. These silica aclosols were labeled SA-1, SA-2, SA-3, and SA-4, according to the concentration of TEOS (the volume fraction of TEOS by adding volume is 6.0%, 11.4%, 16.2%, and 20.5% respectively). In an ice-water bath, three BC wet gels (20 × 20 × 1 mm) were immersed in the alcosols and stirred consistently. The BC wet gel was allowed to soak for a variable period of time to determine the optimal soaking period. The samples were removed from the silica alcosols, dried at 80 °C for 1 h, their average weight was measured. The overall time period range and the number of repetitions of this procedure depend on whether the quality of the dried sample is constant. In this work, this procedure was performed nine times and the overall soaking time period ranged from 20–180 min for each silica alcosol. The optimal period was evaluated based on dried sample weight.

### 2.4. Preparation of the Silica-Bacterial Cellulose Composite Aerogels

The silica alcosols SA-1, SA-2, SA-3, and SA-4 were prepared according to Procedure 2.3. Each silica alcosol with wet BC gels was stirred in an ice-water bath for 3 h, after which the BC wet gels were removed. The composite gels were aged in ethanol at 50 °C for 2.5 h to stiffen the silica gel skeleton and to completely replace the liquid in the wet composite gels with ethanol. The silica-BC composite aerogel (CA) was obtained after the wet composite gel was dried using supercritical CO_2_ fluid (SCF) at 11 MPa and 40 °C, labeled as CA-1, CA-2, CA-3, and CA-4 according to the concentration of TEOS (corresponds to SA-1, SA-2, SA-3, and SA-4 respectively). Photo of the CA-4 was shown in Figure S2.

In addition, after being saturated with silica alcosols which were same as those used to prepare CA-4 (i.e., 6.8 mL TEOS), the thickness of soft wet BC matrices were compressed from 1 mm to 0.6 mm and 0.3 mm with a mechanical property tester at a rate of 1 mm/min, referred to as CA-4/6 and CA-4/3, respectively. When the samples were compressed to the specified thickness, pressure was held for 15 s, then released. The BC matrix without added silica sols was also treated with ethanol solvent exchange and SCF drying, referred to as BM. The nomenclature and partial physical properties of all the samples are given in Table 1.

### 2.5. Characterizations

The density, silica content, porosity, specific surface area, pore-size distribution, thermal conductivity measurements, morphology, and nanostructure and mechanical properties of the samples were measured. The detailed characterization methods are provided in the Supplementary Materials.

## 3. Results and Discussion 

### 3.1. Diffusion of Silica Alcosols in BC Wet Gel

**Figure 2:**
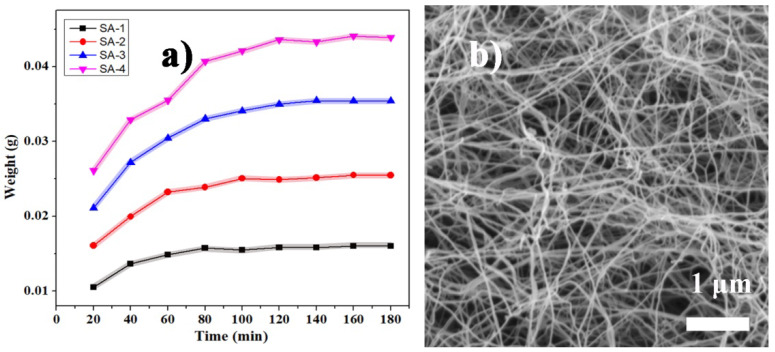
(**a**) The effect of soaking time in the silica alcosols (SA-1, SA-2, SA-3, and SA-4 respectively) on the weight of the dried BC wet gel sample, and (**b**) the microstructure of BC matrix.

The wet BC gel matrix was immersed in the SA-1, SA-2, SA-3, and SA-4 respectively at low temperature (ice-water bath) for varying amounts of time. The weight of the dried samples first increased rapidly, and then the growth rate became gentle and stabilized within 120 min (Figure 2a). This indicates that without condensation in the alcosols, the silica nanoparticles and precursor could diffuse in the BC matrix effectively; a diffusion balance was reached within 2 h. This efficient diffusion was mainly due to the loose microstructure of the BC matrix. The open spaces between the BC nanofibers were often hundreds of nanometers wide (Figure 2b), much wider than the silica nanoparticles, which were only a few nanometers in diameter. This allowed an easy and rapid diffusion of the silica alcosols to occur in the BC matrix. The pores in the BC matrix are much larger and more uniform than the nanoscale pore size of the cellulose nanofibers networks that were used as the matrix in previous studies [44,45,47]. The BC wet gels offered a superior microstructure to the matrix to promote the diffusion of the silica alcosols and the subsequent gelation in situ. Consequently, a soaking period of 3 h was selected to ensure that the silica nanoparticles and precursor were fully diffused in the BC matrix. Additionally, the content of silica in composite aerogels and the density of these samples gradually increased with increasing precursor concentration (Table 1), indicating that the diffusion of silica alcosols was effective and continued to occur at higher concentrations.

### 3.2. Microstructure of the Aerogels

**Figure 3:**
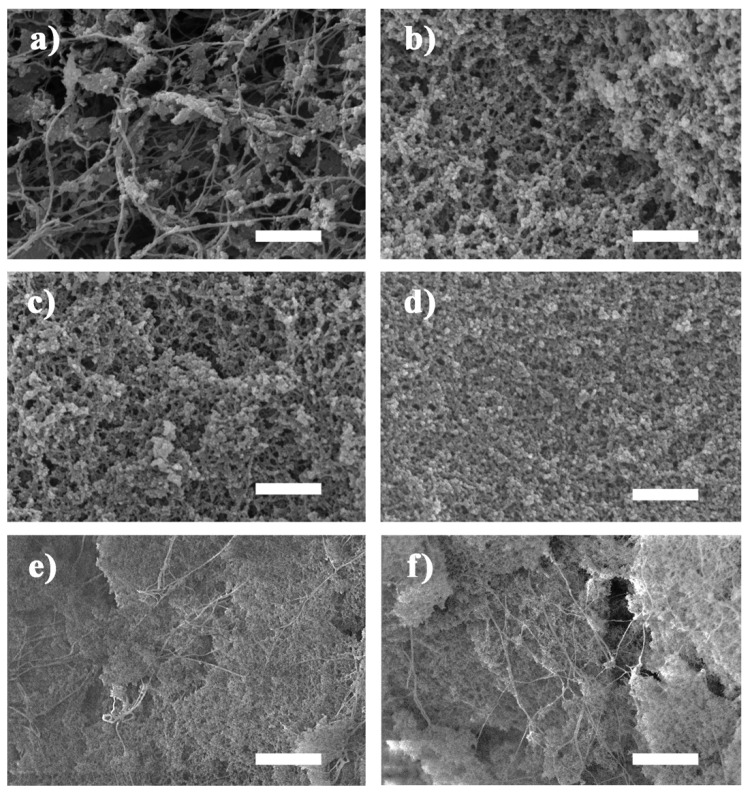
SEM images of (**a**) CA-1, (**b**) CA-2, (**c**) CA-3, (**d**) CA-4, (**e**) CA-4/6, and (**f**) CA-4/3, where the scale bar represents 1 μm.

**Figure 4:**
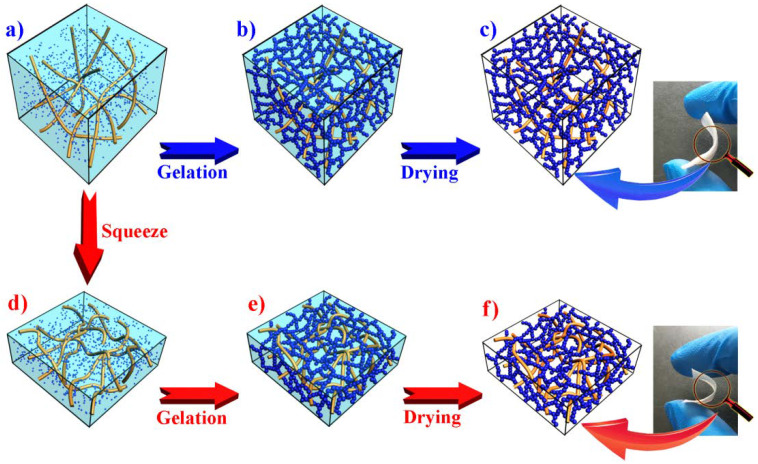
The sol-gel process of silica alcosols in BC matrix before (top) and after (bottom) squeezing some of the silica sols from the matrix respectively. (**a**) and (**d**): BC matrix with silica alcosols; (**b**) and (**e**): BC-silica composite gels; (**c**) and (**f**): composite aerogels. Photographs show the CA-4 (top) and CA-4/3 (bottom) samples.

The scanning electron microscopy (SEM) images of the composite aerogels with varying concentrations of TEOS are given in Figure 3. A silica skeleton formed more effectively in the web-like BC matrix at increased concentrations of the precursor TEOS. Sample CA-1 had the lowest TEOS concentration, and the resulting silica gel skeleton did not fill the entire three-dimensional BC network. Many of the silica gel skeleton agglomerations were attached to the cellulose nanofibers and not connected to one another (Figure 3a). The precursor concentration was too low to form a strong gel skeleton capable of filling a larger space. The cellulose nanofibers (ATR-FTIR spectra are shown in Figure S3) and silica nanoparticles in alcosols were both rich in hydroxyl groups on the surface [48,49,50], and the silica nanoparticles readily gathered around the BC nanofibers to form a silica gel skeleton. An increased amount of silica nanoparticles in the alcosols allowed for the silica gel skeleton to be formed more easily when more precursors were used. The silica gel skeleton also became denser with increased concentrations of TEOS. Furthermore, the microstructure of the composite aerogels was similar to the micromorphology of samples prepared by diffusing the silica alcosols into a dried BC matrix in a previous study by the current authors [37]. This further confirmed that the silica alcosols could penetrate the BC wet gels directly and the matrix did not require prior drying. The CA-4/6 and CA-4/3 exhibited more nanofibers in the field of vision (Figure 3e,f). This indicated that the content of cellulose nanofibers in per unit volume was increased by compressing the matrix containing the silica alcosols before gelation, while the concentration of the precursor in the alcosols and the silica gel skeleton formation remained unchanged by compression (Figure 4). Compared with other composite aerogels toughened with cellulose short fibers [27] or a cellulose nano network [42], the BC nanofibers were more evenly distributed in the gel backbone. In addition, the silica nanoparticles were not attached to the fiber skeleton as they were in Cai’s work [44], but rather, the nanoparticles formed a web-like gel skeleton in the BC matrix.

**Figure 5:**
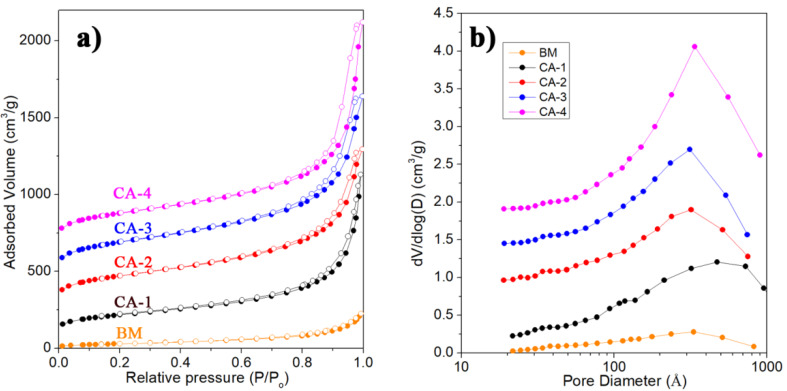
(**a**) Nitrogen adsorption-desorption isotherms and (**b**) BJH pore-size distribution of the BM, CA-1, CA-2, CA-3, and CA-4 samples. It is necessary to illustrate that the curves have been shifted along the Y-axis to show these curves clearly.

The composite aerogels had a stronger adsorption capacity for nitrogen than the BC matrix (Figure 5a). The nitrogen adsorption-desorption isotherms of the CAs exhibited clear hysteresis loops, a classic characteristic of mesoporous materials [24,51], while this was not observed in the BC matrix. Furthermore, the Barrett–Joyner–Halenda (BJH) pore-size distributions of the CAs indicated a distinctive mesoporous structure, while the BC matrix did not (Figure 5b). The specific surface area of CAs was much higher than that of the BC matrix (Table 1), indicating that a mesoporous gel skeleton was formed in the composite silica-BC aerogels. The increased quantity of BC nanofibers with low (113 m^2^ g^−1^) specific surface area in per unit volume showed a slight decrease in the specific surface of CAs, as can be seen for the CA-4/6 and CA-4/3 samples in Table 1. Compared with the silica-cellulose composite aerogels in which only silica nanoparticles were attached to the cellulose nanofiber skeleton, the specific surface area in this work was significantly higher [44]. The width of the peaks in the BJH pore-size distributions became narrower as the concentration of the precursor increased (Figure 5b). Moreover, Figure 5b also shows that with higher concentrations of the precursors that formed the CAs, larger pore volumes in the CAs are obtained (especially mesopores in range 5−50 nm) [52]. Both of these indicated that a higher concentration of precursor resulted in more diffusion of the silica nanoparticles and precursor into the web-like matrix, which aided in the formation of a more uniform porous structure. 

### 3.3. Thermal Conductivity

Compared with sample CA-1, the thermal conductivity of the sample CA-2 becomes higher as the concentration of the precursor increases (Table 1). Insufficient silica gel skeletons were formed in the CA-1 and CA-2 samples, which led to a lack in uniformity. The extreme thermal insulation performance of an aerogel is highly dependent on its mesoporous structure to restrict the movement of gas molecules in the nanopores [53]. Lower integrality and homogeneity of the silica gel skeleton would compromise the distinctive microstructure of aerogels. Instead, the CA-1 and CA-2 samples had a higher content of solid silica as conductive media in the hollow matrix, which resulted in heat conduction. With increased TEOS concentration, an intact and uniform silica gel skeleton was formed that allowed for the structural advantages of aerogels to promote thermal insulation. Hence, the thermal conductivity of CA-3 was lower than CA-1 and CA-2, and the thermal conductivity of CA-4 was only slightly higher than CA-3 as the increased silica gel skeleton allowed for the transport of more heat [53]. Although higher BC nanofiber content resulted in a slight increase in thermal conductivity, the effects were not notable in the CA-4/6 and CA-4/3 samples.

### 3.4. Mechanical Properties

**Figure 6:**
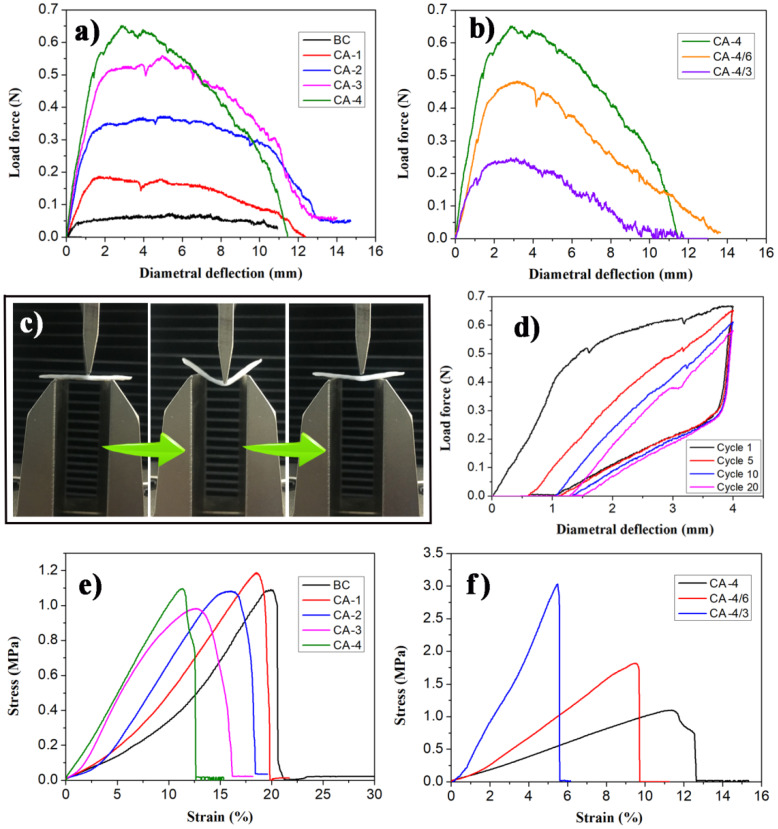
(**a**,**b**) Stress-strain curves of three-point bending tests on all the samples; (**c**) photographs of a three-point bending test on CA-4; (**d**) stress-strain curves of CA-4 under repeated three-point bending tests; and (**e**,**f**) stress-strain curves of tensile tests on all the samples.

All the aerogels were able to withstand a large diametral deformation, i.e., about 10 to 14 mm, without breaking, as demonstrated in the three-point bending tests with a fixture span of 15 mm. This indicated that the CAs were highly flexible. The CAs with a higher concentration of TEOS had a higher maximum flex stress and flexible modulus (Figure 6a and Table 2), demonstrating that a denser silica gel skeleton in the BC matrix increased the material’s resistance to impact from an external force. The maximum flexural stress and flexible modulus of the CA-4/6 and CA-4/3 samples rose sharply as the squeeze degree of the BC matrix increased (Table 2). A larger quantity of BC nanofibers per unit volume caused silica aerogels in different areas to connect more effectively, which strengthened the CAs. Furthermore, CA-4 almost completely returned to its initial shape after 4 mm diametral deformation with a fixture span of 15 mm during durability testing (Figure 6c). The spring back height lost ca. 1.3 mm after 20 cycles (Figure 6d), indicating that the CAs had excellent flexibility and size stability [24]. The excellent flexibility of CAs is further demonstrated in Movie S1. This characteristic of self-recovery after deformation has rarely been reported in other silica-cellulose composite aerogels [27,36,42,43].

The CAs exhibited excellent tensile properties, and the breaking stress of CA-1, CA-2, CA-3, and CA-4 ranged between 0.99 and 1.18 MPa (Table 2 and Figure 6e). These values are similar to the breaking stress of BM, indicating that the robust BC nanofibers in CAs play an important role in resisting tension. As the concentration of the precursor TEOS increased, the fracture elongation of the CAs decreased and the relative tensile modulus gradually increased (Table 2 and Figure 6e). This indicates that a dense and rigid silica gel skeleton in the BC matrix restricted the motion of the BC nanofibers and the gel skeleton under tension. Despite similar densities, the breaking strength and the tensile modulus of CA-4/6 and CA-4/3 were much higher than CA-4 (Table 2 and Figure 6f). More compression of the BC matrix saturated with silica alcosols resulted in a higher breaking strength and tensile modulus because more BC matrix in per unit volume led to more robust CAs that could better withstand the tension. Consequently, the mechanical properties of CAs could be easily controlled through a secondary regulation, such as compressing the matrixes with alcosols.

## 4. Conclusions

Robust and bendable composite aerogels were prepared by directly diffusing silica alcosols into wet BC gel to gradually form an IPN structure in situ. Benefiting from the loose microstructure of the BC nanofibers matrix and the restriction of gelation rate, the silica alcosols effectively diffused in the BC matrix within 120 min, after which a silica gel skeleton was formed in the matrix. In comparison with other methods, the number of steps in the diffusion process of precursor has been reduced to one, and the previous drying of the matrix was found to be unnecessary. The CAs exhibited low density (<0.12 g/cm^3^), high specific surface area (>600 m^2^/g), high porosity (>94%), and low thermal conductivity (<0.034 W m^−1^ K^−1^), as well as excellent flexural and tensile properties. The mechanical properties were easily improved by secondary regulation, which involved compressing the matrixes containing alcosols to increase the quality of BC nanofibers per unit volume of the matrix. This increased the breaking stress and tensile modulus to 3.06 MPa and 46.07 MPa, respectively. The proposed method facilitated the preparation of robust and bendable composite aerogels and exhibits promising potential for improving the development and application of aerogels.

## Figures and Tables

**Table 1:** Physical properties of the dried BC matrix (BM) and composite aerogels (CAs) with varying precursor concentrations.

Materials	SiO_2_ in Aerogels [% *w/w*]	Bulk Density [g cm^−3^]	*S*_BET_^a^ [m^2^ g^−1^]	Pore Volume (cm^3^ g^−1^)	D ^b^ [nm]	P ^c^ [%]	Thermal Conductivity [W m^−1^ K^−1^]
BM	0	0.007	113	0.35	11.4	99.5	0.029
CA-1	78	0.032	440	1.59	14.7	98.4	0.031
CA-2	88	0.060	641	1.50	9.2	97.1	0.034
CA-3	91	0.082	648	1.56	9.5	96.0	0.028
CA-4	93	0.104	667	2.16	13.7	94.9	0.030
CA-4/6	89	0.108	661	2.08	13.2	94.7	0.031
CA-4/3	81	0.119	647	1.96	13.3	94.1	0.033

^a^ BET specific surface area determined using nitrogen sorption. ^b^ Mean pore diameter determined using nitrogen adsorption branch and Barrett–Joyner–Halenda (BJH). ^c^ The porosity includes both mesopores and all void space in CAs.

**Table 2:** Flexural and tensile mechanical properties of the CAs samples.

Materials	Flexural Properties		Tensile Properties		
	Max Flex Stress (MPa)	Flexible Modulus (MPa)	Breaking Stress (MPa)	Elongation at Break (%)	Tensile Modulus (MPa)
BM	0.05	3.45	1.12	20.0	2.95
CA-1	0.21	4.84	1.18	18.3	3.06
CA-2	0.39	9.65	1.09	16.1	2.91
CA-3	0.57	10.99	0.99	12.7	5.42
CA-4	0.73	13.46	1.11	11.5	9.13
CA-4/6	1.47	48.76	1.70	9.6	12.92
CA-4/3	2.88	274.49	3.06	5.4	46.07

Note: Data were obtained from the calculation method described in the Supplementary Materials.

## Supplementary Materials

The following are available online at https://www.mdpi.com/2073-4360/12/4/807/s1, Video S1: video of CA-4 being repeatedly bent and deformed; Figure S1: photos of BC matrix; Figure S2: photo of CA-4; Figure S3: ATR-FTIR spectra of BC matrix and material characterization method.
