# Supplementary material for: Robust Silica-Cellulose Composite Aerogels with a Nanoscale Interpenetrating Network Structure Prepared Using a Streamlined Process
Source: Polymers (Basel). 2020 Apr 3;12(4):807. doi: 10.3390/polym12040807 (PMC7240684; doi:10.3390/polym12040807)
Supplement: Supplementary file 1 [file polymers-12-00807-s001.zip › Supplementary Materials/Supplementary information-Polymers.pdf]

Supplementary Materials (SM)

Robust Silica-Cellulose Composite Aerogels with a Nanoscale Interpenetrating Network Structure Prepared using a Streamlined Process

*Huazheng Sai, Jing Zhang, Zhiqiang Jin, Rui Fu *, Meijuan Wang, Yutong Wang, Yaxiong Wang and Litong Ma*

This Supplementary Materials contains:

1. Supplementary video

2. BC matrix grown in liquid media

3. Photos of CA-4

4. ATR-FTIR spectra of BC matrix

5. Material Characterization

6. Reference

1. Supplementary Movie

Video S1

Video of CA-4 being repeatedly bent and deformed.

2. Photos of BC matrix

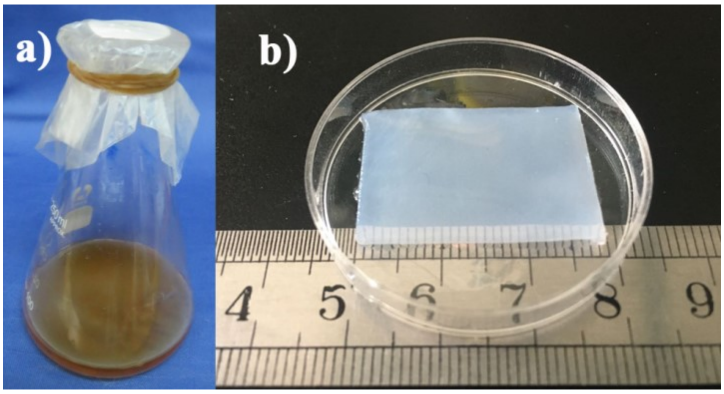

Figure S1. a) the BC matrix grown in liquid media; b) the rectangular BC matrix treated by NaOH.

3. Photo of CA-4

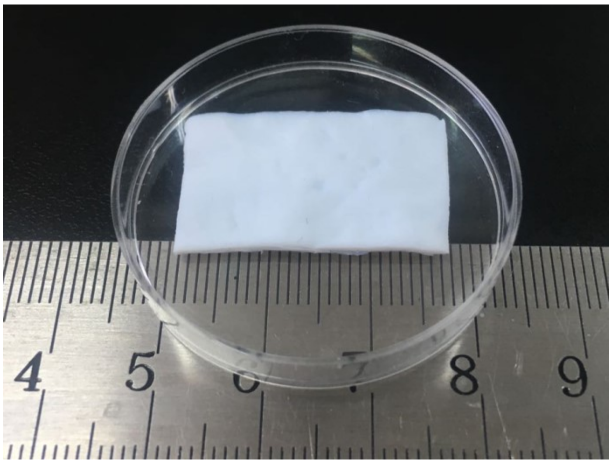

Figure S2. Photo of the CA-4.

4. ATR-FTIR spectra of BC matrix

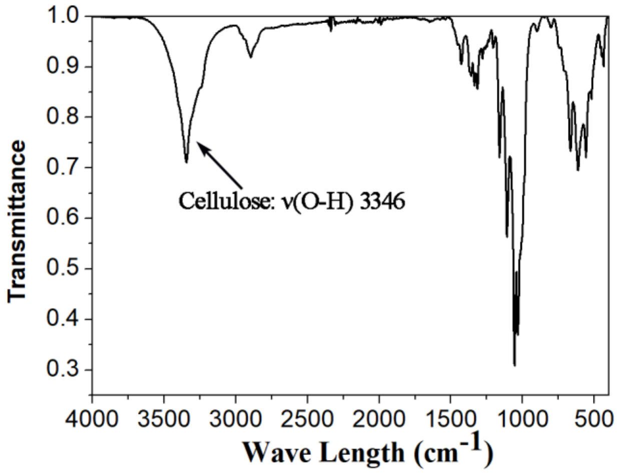

Figure S3. ATR-FTIR spectra of BC matrix after freeze-drying.

5. Characterizations

Nitrogen Physisorption Measurements

First, the sample was degassed at 150 °C for 8 h. Then, the nitrogen physisorption measurements at 77 K were performed with a ASAP2460 (Micromeritics, USA). Brunauer−Emmett−Teller (BET) Analysis from the amount of N_2 absorbed at various relative vapor pressures (eight points $0.05 < p/p_o < 0.2$, nitrogen molecular cross-sectional area = 0.162 nm^2) was used to determine the surface area. The Barrett–Joyner–Halenda (BJH) Analyses were conducted from desorption isotherm when the pore-size distribution was investigated. The pore volume of the samples was determined by the amount of N_2 absorbed at $p/p_o = 0.99$.

Density

The density of BC matrix (BM) and CAs were determined by measuring the weight and volume of each individual sample. The weight of BM and CAs was measured by an analytical balance (readability 0.0001 g, SARTORIUS). Five blocks were used for density determination for each sample.

Content of silica in CAs

The content of silica in CAs was calculated as according to eq 1, where m_s and m_{BC} are the mass of silica and BCnanofibers in the CAs, respectively; ρ and ρ_0 are the bulk density of CAs and BM respectively.

$$Mass\ fraction\ (\omega_s) = \frac{m_s}{m} = \frac{m - m_{BC}}{m} = \left(\frac{\rho - \rho_0}{\rho}\right) \times 100\% \quad (1)$$

Porosity

The porosity of HBM and CAs was calculated according to eq 2, where ρ, ρ_s and ρ_c are the bulk density of CAs, the skeleton densities of pure silica aerogels and BC matrix; ω_s and ω_c were the mass

fraction of silica and BC in CAs, respectively. Herein, based on literature data, the ρ_s and ρ_c were designed as 2.1 g cm^{-3} and 1.59 g cm^{-3} [1, 2].

$$Porosity\ (\%) = \left(1 - \frac{\rho}{\omega_s\rho_s + \omega_c\rho_c}\right) \times 100\% \quad (2)$$

Thermal conductivity measurements

Thermal conductivity of HBM and CAs was measured by a TC3000 thermal conductivity analyzer (Xi'an Xiatech Electronics Co., Ltd.) through a transient hot-wire (THW) method. The sensor was sandwiched between two samples. Then, a weight was put on the sample to make sure the contact between the sample and the sensor plane was complete.

Morphology and Nanostructure

Field-emission scanning electron microscopy (SEM) characterization of the aerogels was performed by TESCAN GAIA3 (Czech) and ZEISS SIGMA 500 (Germany) at accelerating voltage of 10 kV and a working distance of 10 mm. Samples were stuck on the sample holder with a carbon pad and coated with platinum.

Fourier Transform Infrared (FTIR) Spectroscopy

The attenuated total reflection (ATR) infrared spectra of BC matrix was obtained on a VERTEX 70 FT-IR spectrometer (Bruker, Germany). All spectra were recorded between 4000 and 400 cm^{-1} with a resolution of 4 cm^{-1}and 16 scans per sample.

Mechanical Properties

The flexural properties and tensile properties of the sample were measured by a HD-B609B-8 mechanical performance tester (Dongguan Haida Instrument Co., Ltd.). The force was loaded at a speed of 2 mm/min for the tests. The tensile modulus was determined from the slope of the initial linear region of the stress-strain curves. The max flex stress (σ_f) and flexible modulus (E_f) of these samples were calculated according to eq 3 and 4, respectively. In the equations, P is the applied load, L is the sample span, b is the sample width, d is the sample thickness, and Δ is the sample deflection [3].

$$\sigma_f = \frac{3PL}{2bd^2} \quad (3)$$

$$E_f = \frac{L^3 P}{4bd^3\Delta} \quad (4)$$

6. References

[1] Hüsing, N., Schubert, U., Mezei, R., Fratzl, P., Riegel, B., Kiefer, W., Kohler, D., Mader, W. (1999) Formation and Structure of Gel Networks from $Si(OEt)_4/(MeO)_3Si(CH_2)_3NR'_2$ Mixtures ($NR'_2 = NH_2$ or $NHCH_2CH_2NH_2$), Chem. Mater. 11:451-457.

[2] Heath, L., Thielemans, W. (2010) Cellulose nanowhisker aerogels, Green Chem. 12:1448-1453.

[3] Li, L., Yalcin, B., Nguyen, B. N., Meador, M. A. B., Cakmak, M. (2009) Flexible Nanofiber-Reinforced Aerogel (Xerogel) Synthesis, Manufacture, and Characterization, ACS Appl. Mater. Interfaces 1:2491-2501.
